# Overexpression of *Myrothamnus flabellifolia MfWRKY41* confers drought and salinity tolerance by enhancing root system and antioxidation ability in Arabidopsis

**DOI:** 10.3389/fpls.2022.967352

**Published:** 2022-07-22

**Authors:** Zhuo Huang, Li Song, Yao Xiao, Xiaojuan Zhong, Jiatong Wang, Wenxin Xu, Cai-Zhong Jiang

**Affiliations:** ^1^College of Landscape Architecture, Sichuan Agricultural University, Chengdu, China; ^2^Department of Plant Sciences, University of California, Davis, Davis, CA, United States; ^3^Crops Pathology and Genetics Research Unit, United States Department of Agriculture, Agricultural Research Service, Davis, CA, United States

**Keywords:** *Myrothamnus flabellifolia*, drought tolerance, WRKY transcription factor, root system, antioxidation

## Abstract

Myrothamnus flabellifolia is the only woody resurrection plant discovered so far and could recover from extreme desiccation condition. However, few genes related to its strong drought tolerance have been characterized, and the underlying molecular mechanisms remains mysterious. Members of WRKY transcription factor family are effective in regulating abiotic stress responses or tolerance in various plants. An early dehydration-induced gene encoding a WRKY transcription factor namely MfWRKY41 was isolated from *M. flabellifolia*, which is homologous to AtWRKY41 of Arabidopsis. It contains a typical WRKY domain and zinc finger motif, and is located in the nucleus. Comparing to wild type, the four transgenic lines overexpressing *MfWRKY41* showed better growth performance under drought and salt treatments, and exhibited higher chlorophyll content, lower water loss rate and stomatal aperture and better osmotic adjustment capacity. These results indicated that MfWRKY41 of *M. flabellifolia* positively regulates drought as well as salinity responses. Interestingly, the root system architecture, including lateral root number and primary root length, of the transgenic lines was enhanced by MfWRKY41 under both normal and stressful conditions, and the antioxidation ability was also significantly improved. Therefore, MfWRKY41 may have potential application values in genetic improvement of plant tolerance to drought and salinity stresses. The molecular mechanism involving in the regulatory roles of MfWRKY41 is worthy being explored in the future.

## Introduction

As sessile organisms, plants are exposed to constantly varying environmental conditions and will inevitably encounter many abiotic stresses, for example, drought, water logging, heat, cold, salinity, and ultraviolet radiation. To adapt and counteract the negative effects of these abiotic stresses, plants have evolved complex molecular mechanisms involving signal perception, transduction and subsequent protective responses *via* a series of gene expressions (Ma et al., 2019; Yoon et al., 2020).

Member of Transcription factor (TF) families, such as AP2/EREBP (APETALA2/ethylene-responsive element-binding protein), bZIP (basic leucine zipper), NAC (NAM-no apical meristem, ATAF-Arabidopsis transcription activation factor, and CUC- cup-shaped cotyledon), WRKY and MYB (myeloblastosis) have been identified to be involved in abiotic stress tolerance through regulation of stress-responsive gene expression (Agarwal et al., 2006).

The WRKY family is a unique TF superfamily, which play important roles in many biological processes (Yamasaki et al., 2005). The first WRKY transcription factor, the SWEET POTATO FACTOR1 (SPF1), was found in *Impoea batatas* (Ishiguro and Nakamura, 1994). With an increase in available plant genomes, numerous WRKY genes from a wide range of plant species have been characterized, including 45 from *Hordeum vulgare* (Mangelsen et al., 2008), 74 from *Arabidopsis thaliana* (Berri et al., 2009), 81 from *Solanum lycopersicum* (Huang et al., 2012), and 102 from *Oryza sativa* (Ross et al., 2007). The WRKY transcription factor is featured by the WRKY domain comprising of two conserved structures: the N-terminal DNA binding domain composed of a heptapeptide WRKYGQK (WRKY motif) and the C-terminal C2H2 (C-X4(C-C-X22Cin-H-X1-H) or C2HC (C-X7 -C-X23-H-X1-C) types of zinc-finger motif, although different WRKY transcription factors have slight differences in heptapeptide motif and zinc-finger structure (Eulgem et al., 2000; Xie et al., 2005; Huang et al., 2012; Phukan et al., 2016). Based on the number of WRKY domain, WRKY TFs are usually classified to three groups: Group I has two WRKY domains with the C2H2 zinc-finger motif while Group II is the largest group, which has one WRKY domain as same as that of the group I. This group was originally classified into five different subgroups (IIa, IIb, IIc, IId, and IIe). Group III also has only one WRKY domain, but is distinguished from Group I and II by the C2-HC type zinc-finger-like motif (Eulgem et al., 2000). WRKY protein can effectively bind W-box elements to activate or inhibit the transcription of downstream target genes. Moreover, it can also bind other acting elements to form protein complexes, which enhances its activity of transcription binding (Phukan et al., 2016).

According to previous studies, WRKY TFs function effectively in most abiotic stress responses or tolerances in various plants. In response to abiotic stresses, some WRKY TFs can be rapidly induced to promote signal transduction and regulate the expression of downstream stress responsive genes (Jiang and Deyholos, 2009). For example, in *sorghum bicolor, SbWRKY30* regulates the drought stress response gene *SbRD19* by binding with W-box of the *SbRD19* promoter, and protects plant cells from the damage of reactive oxygen species (ROS) by improving ROS scavenging capability (Yang et al., 2020). In addition, WRKY TFs also play essential roles in regulating the response to salinity stress. The *IbWRKY47* gene positively regulates stress resistance-related genes and significantly improves the salinity tolerance of *Ipomoea batatas* (Qin et al., 2020).

*WRKY41* belongs to the group III of WRKY family, and is involved in diverse biological processes such as seed dormancy and plant disease resistance (Higashi et al., 2008; Duan et al., 2018; Lian et al., 2022; Wang et al., 2022). In addition, the role of *WRKY41* in drought and salinity stress was also revealed. For example, Cotton *GhWRKY41* positively regulates salinity and drought tolerances of transgenic tobacco (Chu et al., 2015). Overexpression of *PeWRKY41* improved the insect resistance and salinity tolerance of transgenic *Populus alba* × *Populus tremula* var. glandulosa (Yu et al., 2022).

*Myrothamnus flabellifolia* is native to southern Africa (Bentley et al., 2020). It is highly aromatic and produces a robust profile of secondary compounds related to tolerance and defense. Many of which have important applications (Brar et al., 2018; Bentley et al., 2019). *M. flabellifolia* usually grows in environments with severe abiotic stress (e.g., drought, high temperature, irradiation). Through a long term evolution, *M. flabellifolia* develops strong survival strategies, including developed root systems and the ability to recover from dehydration to adapt to the extremely arid environments. However, due to the lack of genomic resources, genes of *M. flabellifolia* associated with stress tolerance and the underlying mechanisms are largely unknown. A recent tanscriptome investigation of *M. flabellifolia* focusing on dynamic dehydration response showed that a variety of TFs may function in the transcriptional regulatory network during dehydration, among which *MfWRKY41* was significantly up-regulated at the initial stage of dehydration treatment (Ma et al., 2015), suggesting that it may be involved in the regulation of desiccation tolerance. To further validate this speculation, in this study, *MfWRKY41* gene was cloned, and its role in enhancing drought and salinity tolerances was investigated, and its potential regulation mechanism was discussed.

## Materials and methods

### Plant materials and drought and salinity stress treatments

Arabidopsis ecotype *Columbia* (Wild type, WT) is conserved by our lab. *M. flabellifolia* is provided by the Department of Plant Sciences, University of California, Davis. Seeds of transgenic lines and WT were sterilized with 50% bleach solution for 5 min and washed with sterile water for 3–5 times, 1 min each time. Then the seeds were evenly planted on 1/2 MS medium with different concentrations of mannitol (0, 200, 250, and 300 mM). Each treatment was performed in 3 biological repeats, and 15 seeds (plants) for every repeat. After 2-days vernalization in dark and at 4°C, the medium was placed in an illuminating incubator for 10 days at 2510ating incubaced in t. After 2i6 h light/8 h dark. The root length of 15 seedlings of each line (WT or transgenic lines) was measured 10 days after germination, and 3 biological repeats were performed.

WT and transgenic seeds germinated on 1/2 MS medium were planted in substrate (peat soil: vermiculite = 1:1) and grown for 4 weeks at 25°C/23°C (16 h light/8 h dark) and 60% relative humidity. Before the drought treatment, the pots were fully watered and then the watering was hold for several days. For salt treatment, 300 mM NaCl solution was used for irrigation twice per 3-days. The plants under drought and salt treatments were photographed daily. The rosettes of plants treated with natural drought for 8 days and salt (300 mM NaCl) for 12 days were selected for physiological measurement.

### Cloning and sequence analysis of *MfWRKY41*

Plant Total RNA Isolation Kit (Lambo Biotechnology Co., Ltd., Chengdu, China) was used for total RNA extraction. Reverse Transcriptase M-MLV (RnaseH-) (TaKaRa, Dalian, China) was used to synthesize cDNA. Based on sequence of uniGene comp38787_c0_seq2 (Ma et al., 2015), a pair of primers, forward primer: 5′-TCCCCCGGGATGGAGCAGAAGAATTTGAT-3’ (*Sma*I site is underlined) and reverse primer: 5′-GGACTAGTTTA AACTAAGAACTCTGAG-3′ (*Spe*I site is underlined), was designed for gene cloning and vector construction with suitable restriction endonuclease sites according to the binary expression vector pGSA1403. By using High Fidelity Taq DNA Polymerase (TransGen Biotechnology Co., Ltd., Beijing, China), PCR amplification was carried out using *M. flabellifolia* cDNA as template. PCR products with expected size were recovered and purified using agarose gel DNA recovery kit (Tiangen Biochemical Technology Co., Ltd., Beijing, China), then linked to the pEasy-T1 Simple vector (TransGen Biotech, Beijing, China). Subsequently, pEasY-T1-*MfWRKY41* was transformed into *Escherichia coli* strain DH5a. The positive clones verified by PCR were sent to Shanghai Biotechnology Company for sequencing.

BLASTP^1^ was used for homologous gene search, SMART^2^ was used to predict protein domains. DNAMAN 8.0 and MEGA7.0 were used to multiple sequence alignment and phylogenetic analysis, Bootstrap test with 1,000 replications were performed by neighbor-joining method.

### Subcellular localization of *MfWRKY41*

According to the target gene sequence and pHB-YFP expression vector, the primers with appropriate double restriction enzyme sites, *Hin*dIII and *Bam*HI, were designed to amplify the coding sequence of *MfWRKY41* without the stop codon. The primer sequences were as follows: forward: 5′-*ACCAGTCTCTCTCTC*AAGCTTATGGAGCAGAAGAATTT GATCAATG-3′ and reverse: 5′-*GCTCACCATACTAGT*GGA TCCAACTAAGAACTCTGAGCCGTCAAAT-3′ (restriction enzyme sites are underlined and the homologous arm sequences are italic). The expression vector harboring 35S: MfWKY41-YFP fusion fragment was constructed by homologous recombination method using REIII One-step Cloning Mix (Innovagene biotechnology Ltd., Changsha, China). The 35s: MFWKY41-YFP and 35S: YFP (control) were transformed into *Agrobacterium tumefaciens* strain GV3101, respectively. *A. tumefaciens* was injected into wild-type tobacco (*Nicotiana benthamiana*) leaves of 4 weeks old plants. All the transformed tobacco plants were grown in a dark environment of 22°C for 16 h, then moved to normal conditions for 2 days. The fluorescence was observed using laser confocal scanning microscope (Nikon A1, Tokyo, Japan).

### Construction and transformation of binary expression vector

The plasmid DNA of pEasy-T1-*MfWRKY41* was double digested by *Sma*I and *Spe*I (TaKaRa, Dalian, China). T4 DNA Ligase (TaKaRa, Dalian, China) was used to ligate *MfWRKY41* on to binary expression vector pGSA1403 to form pGSA1403-*MfWRKY41*, driven by CaMV 35S promoter. The recombinant plasmid 35S: pGSA1403-*MfWRKY41* was transformed into *A. tumefaciens* strain LBA4404, and Arabidopsis plants were transformed by dip-flower method (Clough and Bent, 1998). The T_1_ seeds were collected and screened on 1/2 MS medium containing kanamycin (50 μg/mL). Kanamycin resistant seedlings were transplanted into soil for selfing. The continuous PCR assay for MfWRKY41 were performed. The T_3_ homozygous positive lines were selected for further Analysis.

### Calculation of water loss rate

WT and T_3_ transgenic plants were cultured routinely for 4 weeks. The plants to be tested were well watered 1 day before the experiment. Approximatively 0.5 g healthy rosette leaves were cut from each strain with scissors and tweezers. Then the leaves were placed in a light incubator with constant temperature and humidity for natural dehydration. The leaves were weighed at 0, 1, 2, 3, 4, 5 and 6 h, respectively, and the water loss rate representing percentage of water loss at each time point can be obtained according to the fresh weights of leaves before dehydration (Qiu et al., 2020).

### Analysis of physiological indexes related to stress

Plant cultivation and sampling were conducted as mentioned above. Leaf chlorophyll was extracted with 95% ethanol (Palta, 1990). Acid ninhydrin colorimetry was used to determine leaf proline content (Bates et al., 1973). Soluble sugar and soluble protein in the leaf were quantified by Plant soluble sugar content detection kit (Nanjing Jiancheng, Nanjing, China) and total protein quantitative assay kit (Nanjing Jiancheng, Nanjing, China), respectively. Histochemical staining with 3,3′- diaminobenzidine (DAB) and nitroblue tetrazole (NBT) was used to visualize the accumulation of hydrogen peroxide (H_2_O_2_) and superoxide anion radical (O_2_^–^) in leaves (Fryer et al., 2002). The leaf malondialdehyde (MDA) content was determined by TBA barbituric acid method. The activities of SOD (superoxide dismutase) and Peroxidase (POD) were measured as previously described (Qiu et al., 2020).

### Stomatal aperture analysis under drought stress

Seeds of WT and transgenic Arabidopsis were sown in nutrition bowl, and cultivated for 4 weeks. Ten healthy rosette leaves of WT and transgenic plants were taken and put into 100 mL MES-KCl buffer for photoinduction for 2.5 h. Five leaves of each line were then taken out from MES-KCl buffer and put into 100 mL MES-KCl buffer containing 300 mM mannitol for light treatment for 2 h. Transparent adhesive tape was used to quickly separate the upper and lower epidermis of leaves. The stomata on the lower epidermal layers of leaf were observed and photographed immediately by optical microscope (DP80, Olympus, Japan). The width-length ratio of 50 stomatal cells of each lines was measured. All experiments were repeated for 3 times.

### Statistical analysis

The research data were analyzed by Student’s *t-*test using SPSS 23.0 software. The measured value is expressed as the mean ± standard deviation (SD) of three repeats, significant differences were considered when *p* < 0.05 (indicated by *) and *p* < 0.01 (indicated by ^**^).

## Results

### Isolation and characterization of *MfWRKY41*

**FIGURE 1:**
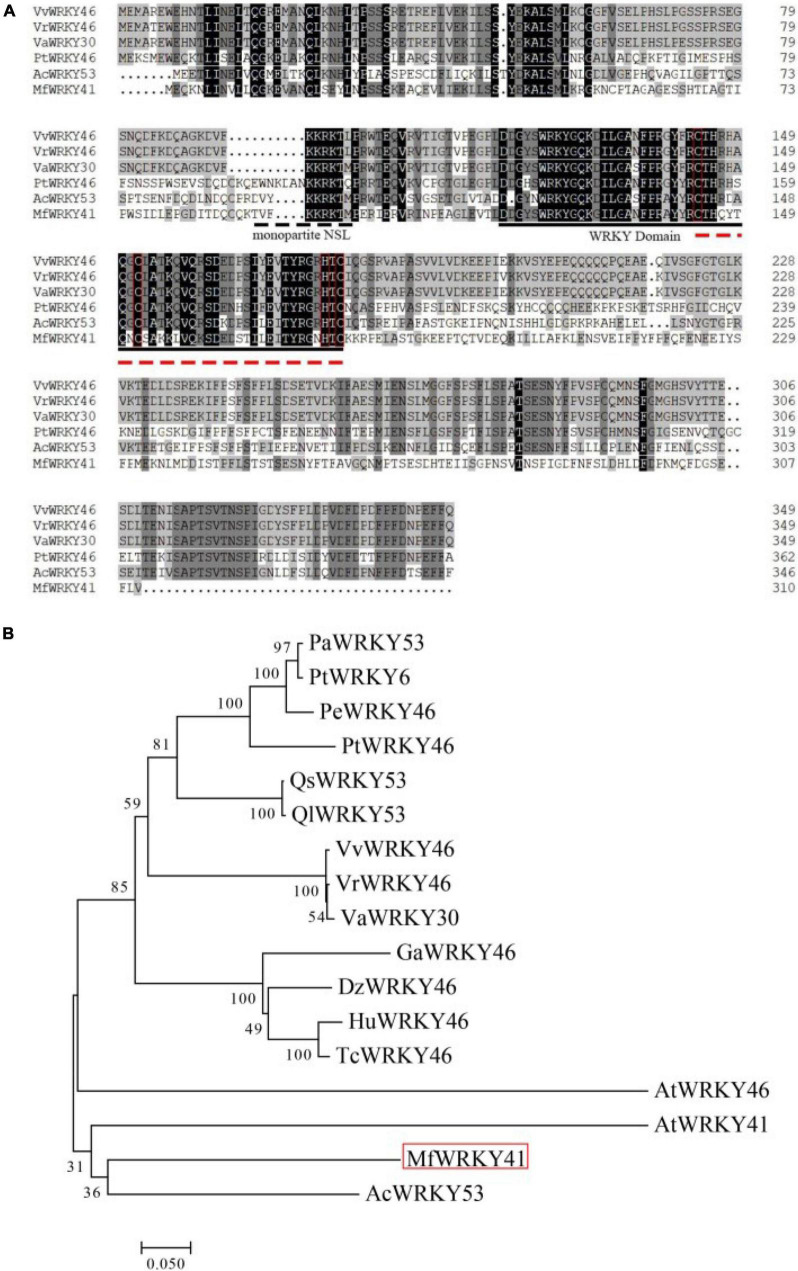
Multiple sequence alignment and phylogenetic analysis of MfWRKY41. **(A)** Black and gray shade showed identical and similar amino acids, respectively. Amino acids marked by the dashed line was the deduced NLS. WRKY domains are represented by solid black line. Zinc finger structure is represented by red dotted lines and red boxes. **(B)** Phylogenetic reconstruction using the neighbor-joining method (Jones-Talor-Thornton model) of MfWRKY41 and several highly homologous WRKY proteins. The MfWRKY41 was marked by red box. The first two letters of sequence name represented initials of Latin name of species from which the sequence originates. The full Latin name and the GenBank accession numbers were listed in Supplementary Table 1.

The cDNA sequence of *MfWRKY41* was cloned from *M. flabellifolia* by PCR amplification. The length of the nucleotide sequence is 933 bp, which possesses an open reading frame (ORF) encoding 310 amino acids. The calculated isoelectric point of MfWRKY41 protein is 12.11, and a predicted molecular mass of 35.6 kDa. A putative monopartite nuclear localization sequence (NLS) “TVFKKRKTMPE” was found (Figure 1A). MfWRKY41 contains a typical WRKY domain, which was composed of 60 amino acid residues. MfWRKY41 also contains a C_*X*7_C_*X*23_H_*X*_C zinc finger motif. Further sequence alignment and subsequent phylogenetic analysis showed that the MfWRKY41 was most homologous to AcWRKY53 of *Actinidia chinensis*, and rather highly homologous to AtWRKY41 and AtWRKY46, respectively (Figure 1B).

### *MfWRKY41* is localized in the nucleus

**FIGURE 2:**
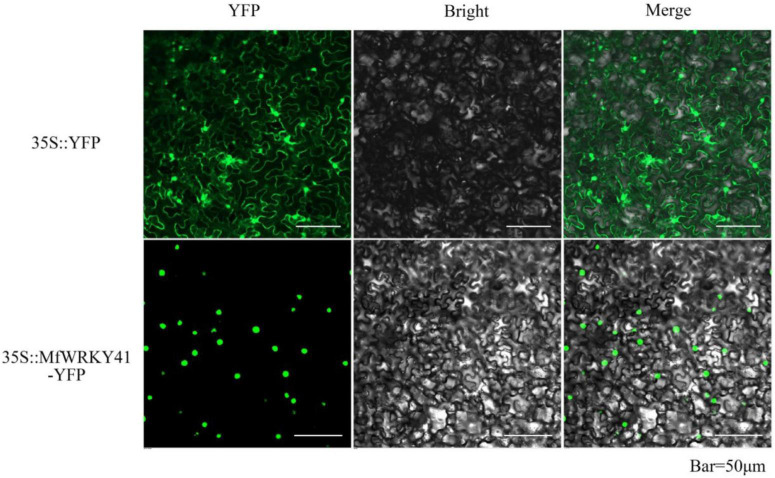
Subcellular localization of MfWRKY41 in tobacco epidermis. Fluorescence detection of MfWRKY41-YFP fusion protein in tobacco leaf epidermal cells, and tobacco epidermis transformed with 35S: YFP was used as a control (upper lane).

A NLS (TVFKKRKTMPE) was predicted within the MfWRKY41 protein (Figure 1A). To confirm the subcellular localization of MfWRKY41, we constructed 35S: *MfWRKY4*1-YFP vector for transient expression in tobacco. Scanning confocal microscope found that the fluorescence of 35S: YFP control group was detected in the whole cells, while strong fluorescence was only observed in the nucleus of 35S: *MfWRKY4*1- YFP transformed cells, which proved that MfWRKY41 is located in the nucleus (Figure 2).

### Overexpressing *MfWRKY41* enhanced tolerance to drought and salinity

To determine the potential function of *MfWRKY41* in abiotic stress response, we constructed a binary expression vector pGSA1403-*MFWRKY41*, with CaMV 35S promotor, to overexpress *MfWRKY41* in Arabidopsis. Kanamycin screening and PCR assays were used to identify positive transgenic lines (Supplementary Figure 1). Four T_3_ homozygous transgenic lines, lines A, C, G, and K, were used for the subsequent analysis.

**FIGURE 3:**
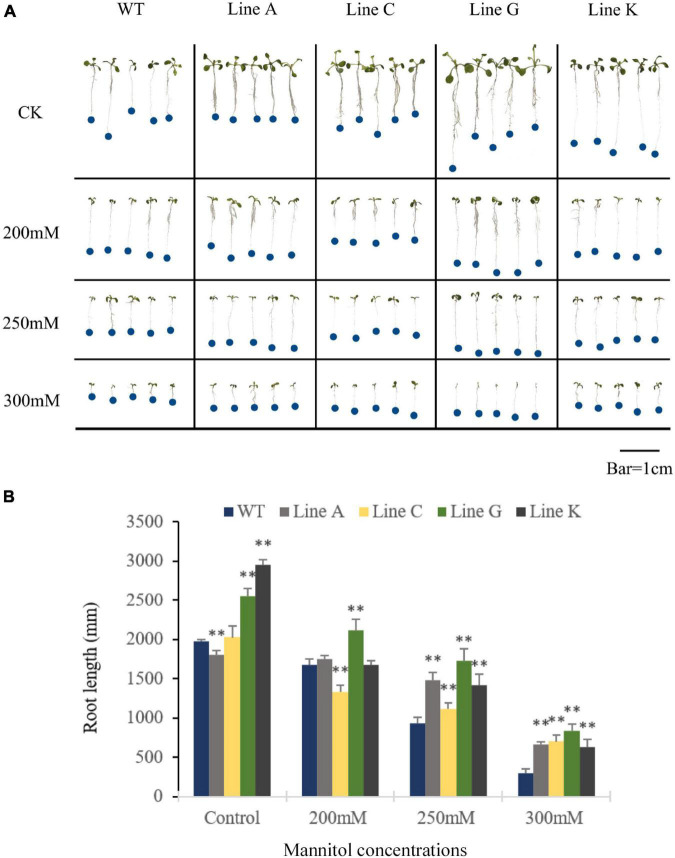
Analysis of drought tolerance at the seedling stage. **(A)** Morphology of transgenic and WT seedlings grown on 1/2 MS medium for 9 days containing varying concentrations of mannitol. **(B)** The primary root length was measured and analyzed. Data are presented as mean and *SD* values of three independent experiments. Asterisks indicated significant difference (^**^P < 0.01) comparing to WT.

In order to investigate whether MfWRKY41 function in drought and salinity tolerances, WT and the four T_3_ transgenic lines were subjected to stress treatment at seedling and adult stages. At seedling stage, the quantity of lateral roots in all transgenic lines was visibly more than WT under normal growth condition, and the root length of line A was slightly shorter, but lines C, G, and K were longer, which were 0.91, 1.03, 1.30, and 1.50 times of that of WT, respectively (Figures 3A,B). With the increase of mannitol concentration, the number of lateral roots apparently decreased; Under treatment of the 200 mM mannitol, transgenic plants, especially lines A, C, and G, also had visible more lateral roots than WT (Figure 3A). Under the treatment of the higher mannitol concentrations (250 and 300 mM), the number of lateral root of both WT and transgenic lines were decreased and not significantly different between each other. Measurement of the primary root length of all lines showed that under the higher concentrations of mannitol (250 and 300 mM), the root length of transgenic lines was 1.20–1.86 times and 2.15–2.80 times of that of WT, respectively (Figure 3B). These results indicated that *MfWRKY41* enhanced root system under artificially simulated drought tolerance.

**FIGURE 4:**
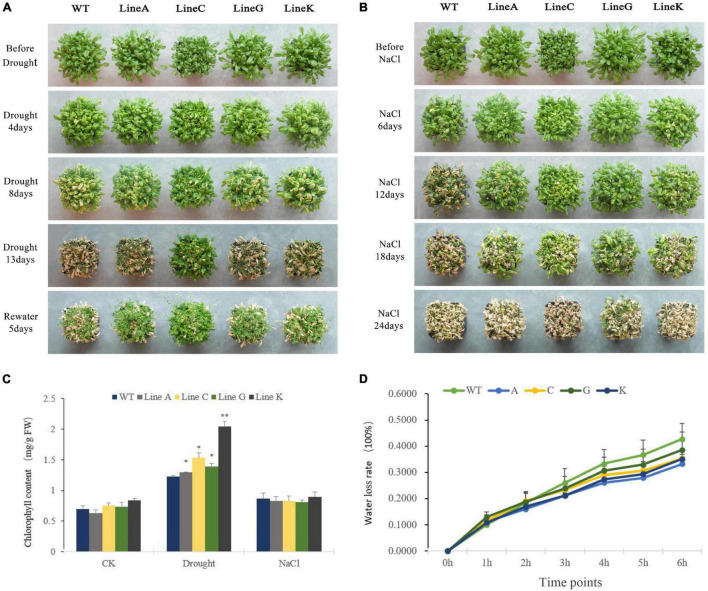
Analysis of drought and salinity tolerance at the adult stage. **(A,B)** Showed the growth status of transgenic and WT plants during natural drought and salinity treatments (300 mM NaCl solution). **(C)** Chlorophyll content of WT and transgenic lines under different conditions. **(D)** Water loss rates of detached leaves measured at 25°C room temperature. Data are presented as mean and *SD* values of three independent experiments. Asterisks indicated significant difference (*P < 0.05, ^**^P < 0.01) comparing to WT.

For the adult stage treatment, all plants were cultivated in soil for 4 weeks under normal condition, and then the natural drought treatment (withholding watering) and salinity treatment (irrigated with 300 mM NaCl solution) were performed. For the drought treatment, we adopted two planting methods: high density with 30 plants per pot and low density with four plants per pot (Figure 4A and Supplementary Figure 2). There was no obvious morphological difference between transgenic plants and WT plants under normal condition, excepting that the line C had slightly smaller shoot than other lines. Under high density cultivation, slight withering was found on all lines at 4 days of treatment; At 8 days of drought, WT showed higher degree withering than those of four transgenic lines; At 13 days of drought treatment, all lines were severely withered, but the transgenic lines, especially line C exhibited more green parts than WT; 5 days after rewatering, all lines can be recovered to varying degrees, and all transgenic lines showed better recovery than the WT (Figure 4A). Under low density cultivation, significant withering could be found at 12 days after withholding watering; At 18 days, all plants were highly withered, however, the transgenic lines especially line C maintained more green parts than that of WT; 5 days after rewatering, three of four transgenic lines, lines A, C, and K, were recovered, but all plants of WT died (Supplementary Figure 2).

The high density planting was used for salt treatment. Clear leaf chlorosis could be found on WT and transgenic lines at 12 days of treatment using NaCl solution, and that in the former was apparently serious than those in the latter; From 12 to 18 days of treatment, all four transgenic lines showed better growth performance than the WT; At 24 days, all plants were extremely withered, but line G still had more green leaves (Figure 4B). All above results suggested that overexpression of *MfWRKY41* enhanced tolerance to natural drought and salinity stress under adult stages.

We measured leaf chlorophyll content of WT and transgenic lines. Under drought treatment, chlorophyll contents of transgenic lines were 1.05–1.66 times higher than that of WT, whereas those under salt treatment were not significantly different (Figure 4C). We further measured the dynamic water loss rate (WLR) of rosette leaf under dehydration. At early stages of dehydration (0–2 h), the WLR of transgenic plants and WT were all most same; From 4 to 6 h, the mean WLR of all transgenic lines were 0.76–0.92 times lower than that of WT, however, the difference was not statistically significant (Figure 4D).

**FIGURE 5:**
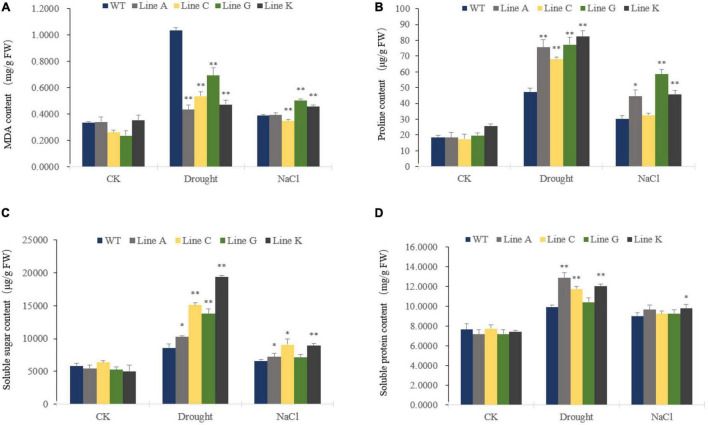
Analysis of membrane lipid peroxidation products and osmotic regulation substances under different stress treatments. Malondialdehyde (MDA) **(A)**, Proline (Pro) **(B)**, Soluble sugar **(C)**, and Soluble protein **(D)** contents were measured for transgenic and WT plants. Data are presented as mean and *SD* values of three independent experiments. Asterisks indicated significant difference (*P < 0.05, ^**^P < 0.01, by Student’s *t*-test) comparing to WT.

Malondialdehyde (MDA) is a naturally occurring product of membrane lipid peroxidation and the MDA accumulation is therefore usually used as a parameter to evaluate the oxidation damage to plant cells under stressful conditions. In our study, MDA levels increased significantly under drought and salinity stresses in transgenic and WT plants. Under drought stress, all four transgenic lines accumulated less MDA, which was 0.42–0.67 times of that of WT. Under salt treatment, transgenic lines showed diverse MDA content: Line A and C showed similar and slightly lower MDA contents than that of WT, respectively, whereas those of lines G and K were 1.30 times and 1.18 times of than the WT (Figure 5A).

Plant cells are prevented from dehydrating by *in vivo* osmotic agents, which improve their tolerance to environmental stress. Therefore we compared the contents of three endogenous osmolytes, proline, soluble protein, and soluble sugar, of four transgenic lines with WT plants. The contents of the three osmotic agents were similar in WT and transgenic lines before treatment (CK). Both drought and salinity stresses increased accumulation of osmotic agents. Under drought treatment, contents of three osmolytes, proline, soluble sugar and soluble protein of transgenic lines were 1.45–1.75 times, 1.19–2.25 times, and 1.05–1.30 times of those in WT, respectively. Under salt treatment, the proline and soluble sugar contents of four transgenic lines were 1.08–1.94 times, and 1.07–1.37 times of those in WT, respectively, in which lines A, G, and K, and lines A, C, and K showed significantly higher content of proline and soluble sugar, respectively. The transgenic lines and WT showed similar content of soluble protein, in which the later was 1.02–1.09 times of that of WT (Figures 5B–D).

### Effect of *MfWRKY41* overexpression on antioxidant metabolism in Arabidopsis under drought and salinity stresses

**FIGURE 6:**
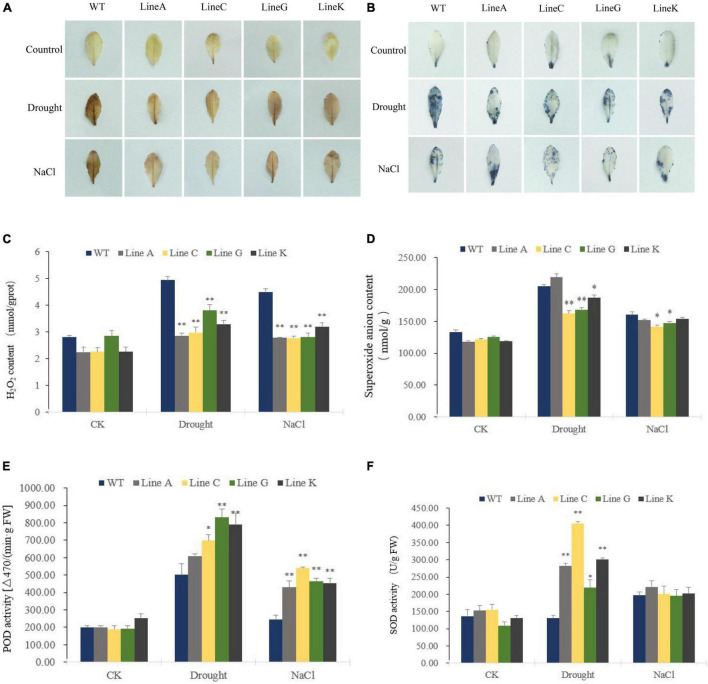
The effect of drought and salt treatments on reactive oxygen species (ROS) content and antioxidant enzyme activities in the *MfWRKY41* transgenic lines and WT plants. H_2_O_2_ and O_2_^–^ were detected by histochemical staining with DAB **(A)** and NBT **(B)**, respectively. **(C,D)** Showed the changes in hydrogen peroxide (H_2_O_2_) content and superoxide anion content between transgenic and WT plants prior to and after stress treatments. **(E,F)** Indicated activities of peroxidase (POD) and superoxide dismutase (SOD) in the leaves of transgenic and WT plants, respectively. Data are presented as mean and *SD* values of three independent experiments. Asterisks indicates significant difference (*p < 0.05; ^**^p < 0.01) comparing to WT.

When plants are exposed to abiotic stress, lipid peroxidation increases along with cell oxidative damage, which is caused by a large amount of excessive reactive oxygen species (ROS) in plant cells, such as hydrogen peroxide (H_2_O_2_), superoxide anion radical (O_2_^–^). To determine the levels of ROS in cells under drought and salinity stresses, we used DAB and NBT histochemical staining. Under normal condition (CK), the leaves were stained in very light brown or few leaf parts were stained in blue. After treatments of drought and salinity, the leaves of WT were dark brown stained by DAB, but the leaves of four transgenic lines were only stained to light brown; By using NBT, the almost whole leaf of WT could be stained in blue under drought, whereas the leaves of transgenic lines were less stained; Under salinity stress, WT was darker stained than those of lines C, G, and K, but in similar staining degree with line A (Figures 6A,B). These results suggested that transgenic lines were suffered from less oxidative damage than WT did.

We further quantified contents of H_2_O_2_ and O_2_^–^ in leaves. They were not significantly different under normal condition. Excepting for line A, H_2_O_2_ and O_2_^–^ contents were significantly higher in the transgenic lines with 0.58–0.76 times and 0.79–0.91 times of those of WT, respectively (Figures 6C,D). Under salt treatment, all four transgenic lines showed significantly lower H_2_O_2_ content, which was 0.62–0.71 times of that in WT, and significantly lower O_2_^–^ content was accumulated in lines C and G, which was 0.88 times and 0.92 times of that in WT, respectively (Figures 6C,D). These results were consistent with those obtained by histochemical staining. The activities of antioxidant enzymes, peroxidase (POD) and superoxide dismutase (SOD), were measured. The two enzymes are responsible for scavenging stress induced H_2_O_2_ and O_2_^–^, respectively. Under drought stress, both POD and SOD activities of transgenic lines were higher than those of WT, which were1.21–3.10 times and 1.21–1.66 times of WT, respectively. Under salt treatment, POD activities of transgenic lines were significantly higher than that of WT, which were 1.75–2.19 times of WT. No significant difference of SOD activities was found between WT and all four transgenic lines (Figures 6E,F). These results were consistent with quantification of H_2_O_2_ and O_2_^–^ content, indicating that transgenic plants are able to reduce cellular oxidative damage in stressful conditions by increasing ROS clearance capacity.

### *MfWRKY41* overexpression promoted drought-induced stomatal closure

**FIGURE 7:**
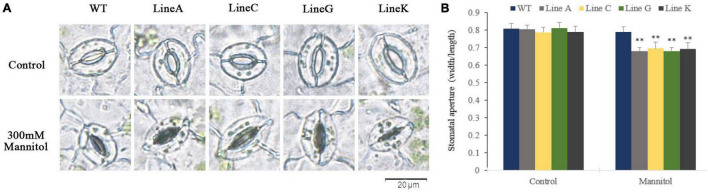
Measurements of the stomatal aperture. **(A)** Microscopy observation of stomatal closure of WT and transgenic lines without (control) and with mannitol treatment (300 mM). **(B)** Measurement of stomatal aperture for transgenic and WT plants. Data are presented as mean and *SD* values of three independent experiments. Asterisks indicated significant difference (^**^textitP < 0.01, by Student’s *t*-test) comparing to WT.

Stomatal movement is essential for plant to survive from drought by regulating transpiration. In this study, we examined the stomatal closure of leaves in the presence of 300 mM mannitol. Under normal conditions, the stomata of all plants are mostly open (Figure 7A), and no significant difference on the stomatal aperture ratios was found between transgenic and WT plants (Figure 7B). With the presence of 300 mM mannitol, all transgenic lines exhibited lower stomatal aperture, which were 0.86–0.88 times of that of WT.

## Discussion

### *MfWRKY41* of *Myrothamnus flabellifolia* improved drought and salt tolerance of Arabidopsis

Members of WRKY TF family have been reported to play important roles in plant responses to abiotic stresses (Li W. et al., 2020). However, few stress-responsive WRKY TFs have been characterized in *M. flabellifolia*, a resurrection plant which can survive from extremely dehydration environment. WRKY41 belongs to group III of WRKY family. Its functions have been investigated previously. Ding et al. (2014) reported that *WRKY41* of Arabidopsis can directly regulate expression of *ABI3* to controls seed dormancy; Overexpression of cotton *GhWRKY41* conferred salt and drought stress tolerance in transgenic tobacco (Chu et al., 2015). Apple *MdWRKY41* was involved in regulation of anthocyanin and proanthocyanidin biosynthesis (Mao et al., 2021); Tomato *ShWRKY41* plays a positive role in resistance to powdery mildew (*Oidium neolycopersici*) (Lian et al., 2022). These reports suggest that *WRKY41* may be multifunctional or have different functions in different organisms.

In this study, we cloned a dehydration-induced WRKY gene *MfWRKY41* from *M. flabellifolia.* To further examine its potential functions in drought and salinity tolerance, we overexpressed it in the model plant Arabidopsis. Four transgenic lines were used in drought and salt treatments at both seedling and adult stages. Our results showed that under either treatment, transgenic lines overexpressing *MfWRKY41* exhibited better growth performance than the WT did (Figures 3, 4), indicating that MfWRKY41 positively regulates the tolerance of plants to drought and salinity.

Root growth changes are important measures for plants to cope with abiotic stresses (Fenta et al., 2014; Awad et al., 2018). At the seedling stage, three of the four transgenic lines have longer primary roots and apparently more lateral roots under normal condition and low concentrations of mannitol stress (Figure 3A). Under higher concentrations of mannitol stress, both primary root length and lateral root numbers were significantly decreased in WT and transgenic lines, but the primary root length of all transgenic lines was significantly longer than that of WT (Figure 3B). These results suggested that overexpression of *MfWRKY41* enhanced root system architecture of transgenic plants, which is different from *GhWRKY41* (Chu et al., 2015), but similar to *MdWRKY30* (Dong et al., 2020) and *FtWRKY46* (Lv et al., 2020).

Previous study reported that inhibition of lateral root growth by drought stress is an adaptive response. Drought or any other abiotic stresses decrease photosynthesis and hence reduce the amount of metabolites and energy. Restriction of the horizontal proliferation of lateral roots in the topsoil and allocation of more resources to the growth of primary roots would offer an advantage to the plants by expanding their domains of water supply (Xiong et al., 2006). Thus, the decreased lateral root number and promotion of primary root elongation under mannitol treatment by overexpression of *MfWRKY41* may increase Arabidopsis’s adaption to drought stress.

### Physiological changes of plants overexpressing *MfWRKY41* under different stresses

Plants can respond to drought and salinity stresses from physiological and biochemical levels, such as photosynthetic physiology, osmotic regulation, scavenging of reactive oxygen species, and plant hormones. Under stress conditions, plant photosynthesis will be inhibited in many aspects, among which the destruction of photosynthetic structure is one of the most important factors, and the amount of chlorophyll content greatly affects the level of photosynthesis efficiency under stress (Fu et al., 2020). Previous studies showed that chlorophyll content in plants tends to decrease due to inhibition of chlorophyll synthesis and oxidative damage under stress (Hanci and Cebeci, 2014; Taïbi et al., 2016). Thus chlorophyll content can be used as one of the indexes to measure drought tolerance of plants. Arabidopsis overexpressing *FtWRKY46* (Lv et al., 2020) and *MxWRKY55* (Han et al., 2020) has higher chlorophyll content and increased salinity tolerance. According to our results, the chlorophyll content of transgenic lines was significantly higher than WT under drought, which was consistent with the phenotypic results of adult plants (Figures 4A–C and Supplementary Figure 2). Therefore, overexpressing *MfWRKY41* in Arabidopsis may enhance drought tolerance by maintaining chlorophyll content to ensure normal photosynthesis.

Accumulation of osmotic regulatory substances is an important indicator reflecting the tolerance of plants (Watanabe et al., 2000; Wang et al., 2005; Gandonou et al., 2011). Overexpression of *FtWRKY46* (Lv et al., 2020), *MxWRKY55* (Han et al., 2020), *IbWRKY2* (Zhu et al., 2020), and *OsWRKY97* (Hou et al., 2020) enhanced tolerance to drought and salinity stresses. The transgenic plants showed higher proline accumulation than that in WT. In this study, we compared accumulations of three osmotic substances under stress, proline, soluble sugar and soluble proteins. Our results showed that the contents of these osmotic substances were generally higher in transgenic lines than those in WT under both stresses (Figures 5B–D). These result suggested that MfWRKY41 may help to regulate osmotic balance under stress.

In plants, stomata are an integral part of gas exchange and water loss. A plant’s ability to maintain water balance and vitality depends on the amount of stomata opening and closing during strong transpiration (Wang and Song, 2008). Under natural drought stress, *MfWRKY41* transgenic plants showed low water loss (Figure 4D), and during simulated drought treatment, transgenic plants also exhibited lower stomatal aperture (Figure 7). These advantages enhanced water retention ability of *MfWRKY41* transgenic plants under water stress.

Excessive ROS produced under adverse conditions will eventually cause oxidative damage to plants (Impa et al., 2012). Plant cells have evolved a variety of antioxidant mechanisms, such as regulating the activities of antioxidant enzymes, SOD, CAT, POD, etc., to eliminate the toxic effects caused by the excessive accumulation of ROS (Li T. et al., 2020; Zhang et al., 2022). Plants overexpressing *SlWRKY28* showed lower H_2_O_2_ concentration and significantly higher ascorbate peroxidase (APX) activity comparing to WT. It was speculated that the regulation of enzyme genes in the ROS clearance pathway induced by *SlWRKY28* was the potential mechanism for transgenic plants to improve their resistance to alkali and salinity (Wang et al., 2020). SOD plays a leading role in scavenging ROS, which can catalyze the disproportionation of O_2_^–^ free radicals, maintain the balance of reactive oxygen metabolism, protect cell membrane structure from damage, and mitigate the effects of water stress on plants to a certain extent (Das and Roychoudhury, 2014). Yang et al. (2019) overexpressed *LpWRKY20* of *lilium microphylla* in tobacco and found that under drought stress, SOD and CAT activities of transgenic plants were higher than those of wild type. In this study, we found that transgenic plants accumulated less ROS than WT, reflecting by lower contents of H_2_O_2_ and superoxide anion (O_2_^–^) under drought and salinity stress (Figures 6A–D). We also found lower MDA accumulation, which is an indicator of lipid peroxidation caused by reactive oxygen species (ROS) (Labudda, 2013; Figure 5A). These results were consistent with the higher activities of SOD and POD activities in transgenic plants, especially under drought stress (Figures 6E,F), indicating that MfWRKY41 enhanced oxidative clearance under stress. Studies on mechanism of how MfWRKY41 enhances root system architecture and antioxidation ability are of interests and worthy being conducted in the future.

## Data availability statement

The original contributions presented in this study are included in the article/Supplementary Material, further inquiries can be directed to the corresponding author/s.

## Author contributions

ZH and C-ZJ designed the experiments. ZH guided the entire study, completed genetic transformation, discussed results, and revised the manuscript. LS and YX participated in function verification. XZ performed phenotyping. ZH and WX participated in data analysis. LS and JW drafted the manuscript. All authors read and reviewed the manuscript.

## Conflict of interest

The authors declare that the research was conducted in the absence of any commercial or financial relationships that could be construed as a potential conflict of interest.

## Publisher’s note

All claims expressed in this article are solely those of the authors and do not necessarily represent those of their affiliated organizations, or those of the publisher, the editors and the reviewers. Any product that may be evaluated in this article, or claim that may be made by its manufacturer, is not guaranteed or endorsed by the publisher.

## Footnotes

1
https://blast.ncbi.nlm.nih.gov/Blast.cgi

2
http://smart.embl-heidelberg.de
