# Supplementary material for: Overexpression of Myrothamnus flabellifolia MfWRKY41 confers drought and salinity tolerance by enhancing root system and antioxidation ability in Arabidopsis
Source: Front Plant Sci. 2022 Jul 22;13:967352. doi: 10.3389/fpls.2022.967352 (PMC9355591; doi:10.3389/fpls.2022.967352)
Supplement: Supplementary file 1 [file Data_Sheet_1.docx]

Table S1. Information of some highly homologous WRKYs used to construct phylogenetic tree in Figure 1b.

| **Genes symbol** | **Species** | **Accession No.** |
| --- | --- | --- |
| *AtWRKY41* | *Arabidopsis thaliana* | OAP01020.1 |
| *AtWRKY46* | *Arabidopsis thaliana* | NP_182163.1 |
| *VvWRKY46* | *Vitis vinifera* | XP_002281031.1 |
| *VrWRKY46* | *Vitis riparia* | XP_034710014.1 |
| *VaWRKY30* | *Vitis aestivalis* | AAR92477.1 |
| *PtWRKY46* | *Populus tomentosa* | AZQ19207.1 |
| *AcWRKY53* | *Actinidia chinensis* var. *chinensis* | PSS03959.1 |
| *QsWRKY53* | *Quercus suber* | XP_023899819.1 |
| *QlWRKY53* | *Quercus lobata* | XP_030966941.1 |
| *PeWRKY46* | *Populus euphratica* | XP_011037871.1 |
| *HuWRKY46* | *Herrania umbratica* | XP_021280339.1 |
| *GaWRKY46* | *Gossypium australe* | KAA3458798.1 |
| *DzWRKY46* | *Durio zibethinus* | XP_022770554.1 |
| *PaWRKY53* | *Populus alba* | XP_034904823.1 |
| *PtWRKY6* | *Populus tomentosa* | APR64475.1 |
| *TcWRKY46* | *Theobroma cacao* | XP_007051596.2 |


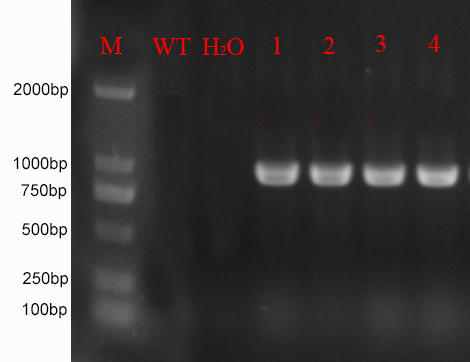


Figure S1 Schematic presentation of PCR identification of positive transgenic lines. M, DNA marker; WT, wild type; 1, 2, 3, and 4 represented transgenic lines A, C, G, and K, respectively.


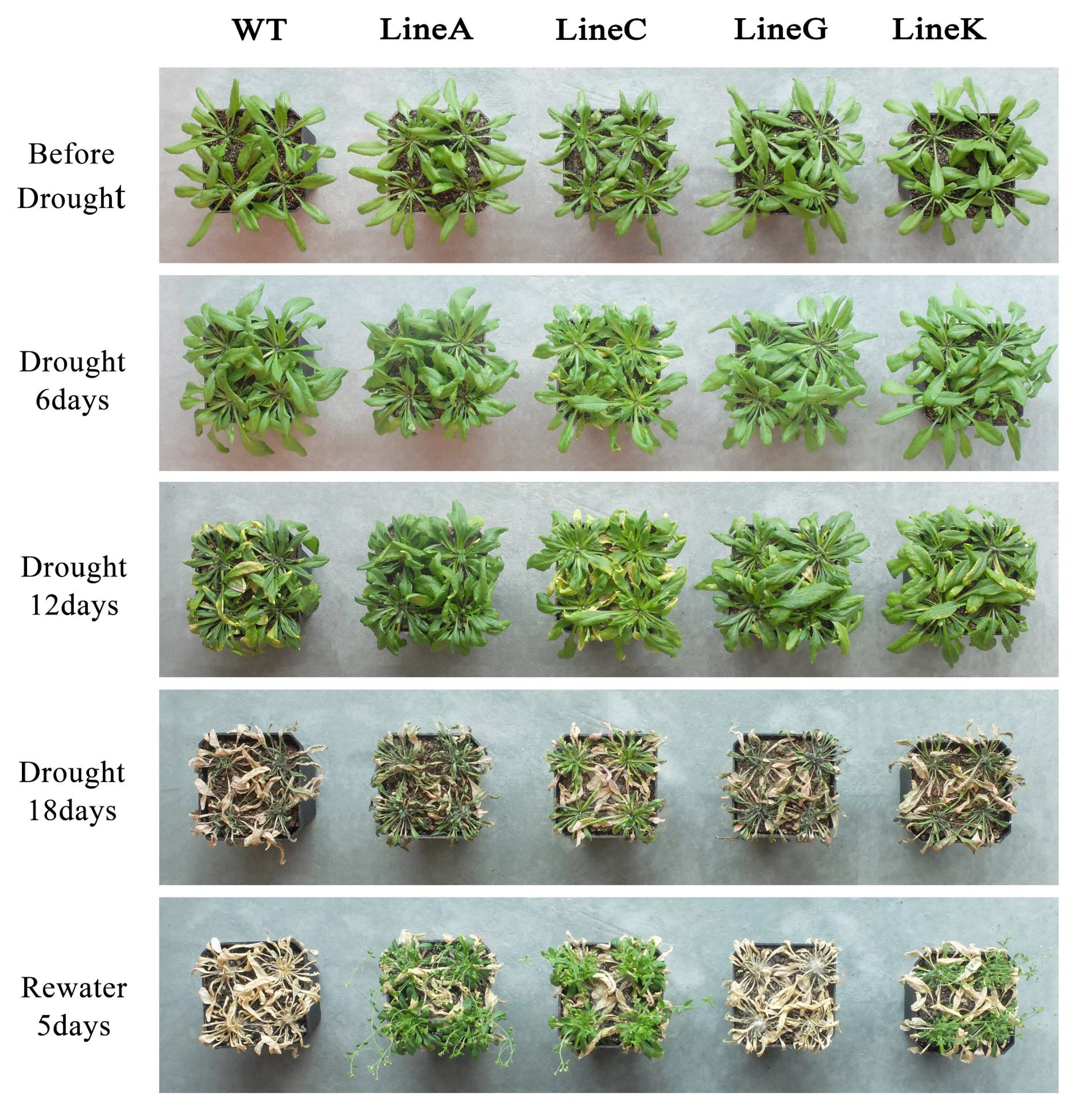


Figure S2 Natural drought treatment under low density cultivation. Four 4-week-old plants with similar growth status were evenly planted in a same pot. The experiments were repeated in three times.
